# People with diabetes need a lower cut-off than others for depression screening with PHQ-9

**DOI:** 10.1371/journal.pone.0240209

**Published:** 2020-10-23

**Authors:** Ewelina Cichoń, Andrzej Kiejna, Andrzej Kokoszka, Tomasz M. Gondek, Rafał Radzio, Adam Jastrzębski, Beata E. Andrzejewska, Fahad D. Alosaimi, Cathy E. Lloyd, Norman Sartorius

**Affiliations:** 1 Department of Psychology, WSB University in Toruń, Toruń, Poland; 2 Department of Psychology, Faculty of Education, Psychology Research Unit for Public Health, University of Lower Silesia, Wroclaw, Poland; 3 II Department of Psychiatry, Medical University of Warsaw, Warsaw, Poland; 4 Specialty Training Section, Polish Psychiatric Association, Wrocław, Poland; 5 Section on Education, World Psychiatric Association, Wrocław, Poland; 6 Institute of Psychotherapy Mind Med Warsaw, Warsaw, Poland; 7 Department of Psychiatry, King Saud University, Riyadh, Saudi Arabia; 8 Faculty of Wellbeing, Education and Language Studies, The Open University, Milton Keynes, United Kingdom; 9 Association for the Improvement of Mental Health Programmes (AMH), Geneva, Switzerland; Universita degli Studi Europea di Roma, ITALY

## Abstract

**Aims:**

This study evaluated the psychometric characteristics of the Polish version of the PHQ-9 in detecting major depression (MDD) and ‘MDD and/or dysthymia’ in people with and without type 2 diabetes.

**Methods:**

Participants were randomly selected from a diabetes outpatient facility (N = 216) and from among patients admitted to a medical center and psychiatric hospital (N = 99). The participants completed the PHQ-9. The Hamilton Depression Rating Scale and the Mini International Neuropsychiatric Interview were used to identify the presence of psychiatric symptoms. The optimal cut-offs for PHQ-9 in people with and without type 2 diabetes were investigated based on two methods: 1) Youden’s index which identifies cut-off points useful in scientific research; 2) a second method of two-stage screening for depressive disorders to provide guidance for clinical practice.

**Results:**

The Polish version of the PHQ-9 is a reliable and valid screening tool for depression in people with and without type 2 diabetes. An optimal cut-off of ≥ 7 was indicated by Youden’s index and ≥ 5 by the two-stage method for screening for MDD and ‘MDD and/or dysthymia’ in the group with type 2 diabetes. A cut-off of ≥ 11 was optimal for screening for both MDD and ‘MDD and/or dysthymia’ among people without diabetes (Youden’s index). The two-stage approach suggested a ≥ 10 score for screening for MDD and ≥ 9 for screening for ‘MDD and/or dysthymia’ in people without diabetes.

**Conclusions:**

A lower cut-off score of the PHQ-9 is recommended for people with type 2 diabetes as compared to the general population.

## 1. Introduction

Depression is particularly common in people with chronic illness and disability, its prevalence among people with diabetes is at least twice as high compared to the general population [1]. The influence of depression on the outcomes of comorbid medical illnesses, such as cardiac diseases, diabetes and cancer, is significant [2].

Multimorbidity is associated with higher health care use and costs, and the severity of depressive symptoms is associated with functional impairment as well as with higher costs of care in people with diabetes [3, 4].

Although both type 1 and type 2 diabetes share the problem of high levels of blood sugar, they are two different diseases in many ways. According to the latest (2020) estimates from the Centers for Disease Control and Prevention (CDC), type 1 diabetes affects just 5 percent of adults with diabetes with type 2 diabetes (T2DM) affecting up to 95 percent [5].

Depression relates mostly to people with T2DM [6]. The co-morbid conditions of depression and T2DM needs a careful attention [7] and treatment must include lifestyle, antidepressant medicines, and psychotherapy [8]. Unrecognized depressive symptoms can lead to less physical activity, difficulties with self-medication and to hyperglycemia [9–11], whereas low levels of physical activity and insufficient self-care lead to poor microvascular and macrovascular outcomes and to an increased risk of mortality [10, 12]. Improving mental health can thus improve clinical outcomes such as metabolic control, and thus reduce the risk of diabetes complications [3, 9, 13–15].

Mood disorders in patients with diabetes often take the form of persistent depressive disorder–dysthymia [16–19]. It has been also shown that about 70% of patients with diabetes and major depression relapsed an average of four episodes over 5 years [20]. Studies have suggested that both dysthymia and minor depression or residual symptoms after a major depression are risk factors for subsequent major depressive episodes [21, 22]. The chronicity of depression in people with diabetes suggests the importance of enhanced screening, treatment of depression and intervention preventing relapses in patients with comorbid depression and diabetes [19].

Indeed, it has been shown that older patients with diabetes and comorbid major depression or dysthymia who receive psychological care were less likely to die over the course of 5 years [23] as well as having less severe depression and greater improvement in overall functioning compared to usual-care practices [24].

Reimer et al. [25] indicated that depression might be more persistent in people with T2DM with elevated depressive symptoms and diabetes-related distress increasing the risk of persistent elevated depressive symptoms. Severe hypoglycaemia was positively associated with the severity of depressive symptoms in Japanese patients with T2DM independent of glycemic control, insulin therapy, lifestyle factors, and diabetic complications [26].

Thus, it is important to screen patients with T2DM not only for Major Depressive Disorder (MDD), but also for depressive symptoms which may not meet the diagnostic criteria for current major depression but may rather take the form of dysthymia. Given the higher risk of depression in people with diabetes, it is essential to routinely screen for depressive symptoms in this group, as prompt provision of appropriate treatment will facilitate the mental and physical condition of patients with comorbidities [2, 15].

Short, simple self-report screening instruments for depression are available, e.g. the Beck Depression Inventory (BDI) [27], the Hamilton Depression Rating Scale (HDRS) [28] and the Patient Health Questionnaire—Nine Item (PHQ-9) [29]. Nevertheless, the recognition of mental health problems such as depression or anxiety does not exceed 50% [29, 30]. Most screening tools have limited capability to discriminate between the overlapping symptoms of mental and somatic disorders [31, 32]. The overlapping symptoms between mood disorders and diabetes, e.g. sleep problems, feeling tired/loss of energy or appetite changes are included in the BDI [27], HDRS [28], and the PHQ-9 [29]. However, these problems are commonly reported by people with diabetes [33–35]. The effective detection of depressive symptoms requires a suitable threshold, i.e. the lowest possible score on a standardized test that a patient must achieve to be considered as having significant depressive symptoms. However, such cut-offs vary depending on the population that is being considered [36]. It is thus possible that there is under-recognition of depression in people with diabetes caused by using inadequate cut-offs, even if these cut-off scores can be useful for people without diabetes. There is thus a need to examine whether these cut-offs might be different in people with diabetes and in addition establish the appropriate balance between sensitivity and specificity for the screening tool in this specific population. Given the partial overlapping of some of the symptoms of diabetes and depression it is possible that the optimal balance between the sensitivity and specificity for people with T2DM may be achieved with a different cut-off score than in healthy people. As shown by van Steenbergen-Weijenburg et al. [37], the cut-off point may be higher in chronically ill patients to correctly identify MDD in the chronically ill than in a population with less severe illnesses [37]. However, depending on the population of patients, there might also be an opposite tendency to neglect the symptoms of depressive disorders and attribute the depressive symptoms to the worsening course of diabetes–by both patients and clinicians. In such cases depression may be overlooked which in turn may lead to further deterioration of glycaemic control.

Given the high prevalence of depressive disorders in people with T2DM and the detrimental interactions between the two disorders, it is suggested that providing the ideal therapy of depression for this population would be an intervention that reduces the symptoms of depression and improves glycemic control concurrently [38]. Therefore it is important to establish a cut-off point which will provide good sensitivity and allow an effective detection of comorbid depressive disorder. The optimal cut-off point may be lower in people with diabetes when compared to the general population, considering the sensitivity of the screening tool is likely to improve with a lower cut-off point.

The recognition of depressive symptoms is crucial in order to provide appropriate health care and the PHQ-9 was developed to fulfill this purpose [29, 39]. The PHQ-9, in comparison with other tools for screening for depressive symptoms, complies with five requirements–it is brief, self-administered, multipurpose, in the public domain, and easy to score [40, 41]. The PHQ-9 has shown adequate reliability and validity in various populations and is widely used as a validated screening tool in primary care [29, 39, 42]. Despite its widespread use and availability in many languages, there is a lack of research evaluating the psychometric properties of the adapted tool, and the English standards are commonly used [43]. This practice does not always allow investigators to achieve the right results.

Using the structured mental health professional interview as the criterion standard, a PHQ‐9 score of ≥ 10 was reported as the optimal cut-off for major depression with 88% sensitivity and 88% specificity in primary care [29] and medical settings (91.7% sensitivity, 78.3% specificity) [39]. The validation of the PHQ-9 for screening for MDD in the general population in Brazil determined a value > 9 as the most optimal cut-off point [44], while among the Chinese general population a score of > 7 was reported when compared with the Mini International Neuropsychiatric Interview (MINI) as the diagnostic standard [41]. In the Polish sample of the general patient population, the sensitivity and specificity of the PHQ-9 in detecting a MDD were 82% and 89%, respectively [42, 43]. The authors reported the best optimal cut-off score of > 12 (≥ 13).

In their very interesting study, Stafford et al. [45] validated the PHQ-9 among cardiac patients in general hospitals. The participants were assigned to two groups, i.e. to MDD or to ‘any depressive disorder’, according to the MINI as the criterion standard. The optimal cut-off score for ‘any depressive disorder’ among cardiac patients is ≥ 5 (81.5 sensitivity and 80.6 specificity), whereas an optimal cut-off score of ≥ 6 was recommended for MDD (sensitivity = 82.9%; specificity = 78.7%). Thus the standard cut-off score of ≥ 10 becomes inappropriate for the recognition of depression in cardiac patients. Also, in the Polish sample for hospitalized elderly patients, a score of > 6 was the optimal cut-off point [46].

It is worth noting that there is evidence to suggest that when screening for depression in diabetes patients in specialized outpatient clinics, a cut-off point of ≥ 12, has been reported to have a sensitivity of 75.7% and a specificity of 80.0% [38]. However, it is important to take into account the characteristics of the population in order to achieve accuracy in depression recognition by physicians, nurses and researchers.

Therefore, the main aims of our study were: (1) to assess both the reliability and validity of the Polish version of the PHQ-9 in patients with and without diabetes; (2) to determine the optimal cut-off point that would indicate a high probability of recognizing a disorder of major depression or of MDD and/or dysthymia, and to verify the hypothesis that the cut-off point is different for the Polish version (for Polish patients) than for the original English version in the general population.

## 2. Materials and methods

### 2.1. Participants

The data reported here pertains to the Polish sample derived from the International Prevalence and Treatment for Diabetes and Depression (INTERPRET-DD) study which was a collaborative study among invited outpatient clinic attendees with T2DM in 14 different countries [47, 48]. The investigators were psychiatrists recruited from leading university centers in Poland. The exclusion criteria were: diagnosis of Type 1 diabetes; diabetes lasting for less than 12 months; incomplete set of measures due to communication and/or cognitive difficulties; any life-threatening or severe conditions, currently admitted or planning admission for inpatient care to a hospital; pregnancy or childbirth in the last 6 months, clinical diagnosis of alcohol or other substance (not tobacco) dependence, or a diagnosis of schizophrenia. A total of 216 Polish individuals with T2DM (101 females, 115 males) took part in this study.

The data for the comparison group is derived from another study carried out by the authors of this paper [43]. The sample consisted of casual selected outpatients at the Department of Internal Diseases, Nephrology and Transplantology of the Central Clinical Hospital of the Ministry of Interior and Administration in Warsaw and of randomly selected outpatients of the Department of Psychiatry at Bródno Hospital. The comparison group was diverse in terms of health and consisted of 99 persons (54 women and 45 men).

### 2.2. Procedure

As part of the INTERPRET-DD study, each eligible individual completed a survey recording his/her age, duration of diabetes, family history of diabetes and presence/history of diabetes complications, any medications for mental health problems or documented diagnosis or treatment of any psychiatric condition(s), the most recent blood pressure measurement, HbA1c, height, weight, location of his/her accommodation (rural or urban area), level of education, marital status, and financial status.

The diagnostic status of all the participants was determined by the MINI Version 5.0.0 in both studies [49, 50]. A written informed consent form was obtained from each participant.

Finally, in both studies the participants completed a set of questionnaires [43, 48] including the same versions of the PHQ-9 [29] and were assessed by a clinician using the HDRS [27, 28]. The original English version of PHQ-9 has been developed by Drs. Robert L. Spitzer, Janet B.W. Williams and Kurt Kroenke. According to the Instruction manual for Patient Health Questionnaire (available at: https://www.phqscreeners.com/images/sites/g/files/g10016261/f/201412/instructions.pdf), the translations have been developed by the MAPI Research Institute using standard forward/back-translation procedures and are linguistically valid. The Polish version of PHQ-9 was downloaded from: https://www.phqscreeners.com (accessed at 19th of July 2020). It has also been validated in the hospitalized elderly population in Poland [46]. In addition, the Polish investigators made sure that the PHQ-9 was culturally applicable through a discussion on the contents of the translated items and by testing them among healthcare professionals and people with T2DM, with a focus on the semantic meanings of the expressions and language used in the questionnaire.

### 2.3. Measures

In order to validate the Polish version of the PHQ-9, relevant data were extracted from the INTERPRET-DD study dataset [47] and from a study by Kokoszka et al. [43]. We took into consideration the occurrence of depression established using the MINI Version 5.0.0 and the participants’ results in the PHQ-9 and HDRS scale, which was used as an external scale to verify the convergent validity of the Polish version of the PHQ-9.

The PHQ-9 is a 9‐item depression module from the full PHQ [29]. This is a self-report screening tool that recognizes the presence and severity of depressive symptoms. The items are based on DSM-IV criteria for the assessment of depressive symptoms during the previous two weeks [29]. Each item of the PHQ-9 scores from 0 (not at all) to 3 (nearly every day), with a summed score ranging from 0 to 27. The severity of a depressive disorder can be assessed as follows: 5–9 (mild), 10–14 (moderate), 15–19 (moderately severe), and 20–27 (severe) [29]. So far, the psychometric qualities of the Polish version of the PHQ-9 in the diabetes population have not been examined.

The HDRS [28] is a commonly used tool to assess the severity of depression symptoms administered by interview and observation. The patient is rated by a clinician according to the specified criteria on a scale from 0 to 4. The most commonly used 17-item version of the HDRS was employed in this study.

The diagnostic status of all the participants at the time of the PHQ-9 assessment was determined by the MINI Version 5.0.0 [49, 50], which has been widely used among different populations, including those with serious illnesses. It is a reliable diagnostic instrument according to Diagnostic and Statistical Manual of Mental Disorders 5th edition (DSM-5) criteria [49]. MDD was diagnosed when participants fulfilled at least one core criterion of the DSM-IV (depressed mood or loss of interest/pleasure) and required additional criteria with a 2-week duration almost every day/night: significant weight loss (or poor appetite) or weight gain; insomnia or hypersomnia; psychomotor retardation/fatigue/loss of energy; feelings of worthlessness/guilt; diminished ability to think/concentrate, indecisiveness; recurrent thoughts of death/suicidal ideation, plan, or attempt. Dysthymia was diagnosed if participants felt sad, low or depressed most of the time for the last two years and fulfilled at least two additional criteria in the past 2 years: weight loss (or poor appetite)/weight gain; insomnia or hypersomnia; fatigue or loss of energy; low self-esteem; diminished ability to think or concentrate, or indecisiveness; feelings of hopelessness. For comparison, participants were classified to the ‘major depressive disorder’ group, or to a more general category, i.e. ‘MDD and/or dysthymia’. The second group consisted of participants who fulfilled the diagnostic criteria of current major depression or/and dysthymia according to the MINI 5.0.0.

### 2.4. Statistical analyses

#### 2.4.1. Internal consistency reliability and convergent validity of the Polish version of the PHQ-9

The statistical analyses were carried out using SPSS version 25 for Windows. In order to determine the internal consistency reliability of the PHQ-9, Cronbach’s alpha was conducted with α values between 0.80 and 0.90 usually indicating good internal consistency [51].

Pearson product-moment correlations were applied to measure convergent validity. We assumed that the PHQ-9 scores would be positively associated with the HDRS. A strong or moderate strength of the relationship (*r* value from |0.50| to |0.80|) between the PHQ-9 scores and the HDRS indicates satisfactory convergent validity [52].

#### 2.4.2. Screening accuracy for likely depression

Logistic regression was performed to assess the discriminatory validity of the Polish version of the PHQ-9 as a screening tool for current ‘MDD and/or dysthymia’ in the general population and in those with T2DM separately for these two groups. A positive predictive value (PPV) and negative predictive value (NPV) were calculated using logistic regression. The PPV is the probability of disease for positive test results, whereas the NPV means the probability of being healthy when the test results are negative [53, 54]. Then we employed the Wald statistic to test whether the PHQ-9 score is a significant predictor of depression. Odds ratios (ORs) and their confidence intervals (CIs) were estimated.

Criterion validity was investigated by computing sensitivity and specificity for all the cut-off scores on the PHQ-9 for MDD and separately for MDD and/or dysthymia diagnoses based on the MINI as the criterion standard. To determine sensitivity and specificity of the PHQ-9, the Receiver Operating Characteristic (ROC) curve was mapped and the area under the curve (AUC), as an effective measure of accuracy of the PHQ-9 for identifying ‘major depressive disorder’ and ‘MDD and/or dysthymia’ for the two groups, was calculated. In most of the previous studies, researchers did not report the applied criteria for the choice of the optimal cut-off [29, 39, 41, 43, 46]. We identified the optimal cut-off values in one step using Youden’s index (sensitivity+specificity−1), which ranges between 0 to 1, with higher values indicating greater diagnostic performance [55]. We also used a second method of two-stage screening for depressive disorders. According to this approach, cut-off scores demonstrating maximal sensitivity and specificity ≥ 75% are recommended [45, 56]. The two-stage approach is more appropriate in clinical settings where positive screening results are usually further verified with a diagnostic interview, observation and treatment [45]. The one-stage method is more suitable in research studies where the results are used to estimate depression prevalence rates and do not lead to clinical decisions [56]. It is worth mentioning that if we used screening to assess if the study’s eligibility criteria were met, a two-stage approach would be more appropriate [45]. Statistical significance for all of the conducted analyses was established at *p <* .05.

## 3. Results

### 3.1. Demographic, clinical and psychological sample characteristics

The demographic, clinical, and psychological characteristics of the participants are presented in Table 1.

**Table 1:** Socio-demographic characteristics and comparison of the group of patients with type 2 diabetes (N = 216) and control group (N = 99).

**Demographics**		**Control Group (N = 99)**		**Patients with T2DM (N = 216)**		**Statistics**
		**n(%)**^a^		**n(%)**		
**Gender**	Females	54(54.5%)		101(46.8%)		χ2(1) = 1.647, p = .199
	Males	45(45.5%)		115(53.2%)		
		χ2(1) = 0.818, p = .366		χ2(1) = 0.907, p = .341		
		M (SD)	M_rank_	M (SD)	M_rank_	
**Age**		41.77 (12.50)	77.80	57.53 (7.18)	194.76	U = 2752.50, p < .001
**Diabetes duration**		9.45 (7.09)	-	-	-	

Note:

a–percent of outpatients within sub-group

Quantitative data (e.g. age) were presented in the form of mean (*M*) and standard deviation (SD). To test whether gender and diabetes were independent the chi-square test of independence was used. The result indicates that diabetes is does not associated with gender, χ^2^ (1) = 1.647, *p* = .199. The one-sample chi-square test was used to verify whether a gender variable follows a hypothesized population distribution. Both in the control group (χ2(1) = 0.818, p = .366) and group of patients with T2DMs (χ2(1) = 0.907, p = .341) the gender ratios were consistent with expected distributions. The comparisons of age between control group and patients with T2DM were conducted using a nonparametric Mann–Whitney U test because of unequal sample size. Thus, the rank mean was presented. The result indicated that the age of control group was significantly lower than age of patients with T2DM, *U* = 2752.50, p < .001 (see Table 1).

PHQ9 scores were not significantly associated with age in either the group of people with T2DM, (*r* = -0.103, *p =* .130) or in the control group (*r* = 0.065, *p =* .521). There was no difference in PHQ-9 scores with regard to either gender (*t*(214) = 1.79, *p =* .075) or education level (*H*(2) = 2.925, *p =* .232) in the group of patients with T2DM. PHQ-9 scores were also not significantly associated with diabetes duration (*r* = 0 .07, *p =* .279).

The comparison group was diverse in terms of health and consisted of 99 persons (54 women and 45 men). PHQ-9 scores in this group were not significantly associated with age (r = 0.065, p = 0.521). There was no difference in results of PHQ-9 with regard to gender (*t*(97) = -0.569, *p =* .57) (see Table 2).

**Table 2:** Associations between participants’ demographic and clinical characteristics with PHQ-9 scores.

**Participants with diabetes**				
		PHQ-9 scores *M* (SD)	*statistic*	*p value*
Gender				
	Male (n = 100)	3.93 (4.87)	*t*(214) = 1.79	= .075
	Female (n = 116)	5.17 (5.29)		
Total sample (N = 216)		4.51 (5.10)		
Age		57.53 (7.18)	*r* = -.103	= .130
Education level				
	No formal (n = 0)	-	*H*(2) = 2.925^a^	= .232
	Some/completed primary school (n = 19)	4.58 (4.65)		
	Some/completed secondary school (n = 140)	4.97 (5.53)		
	Higher education (college, post-grad/professional) (n = 57)	3.35(3.88)		
Diabetes duration		9.45 (7.09)	*r* = .074	= .279
**Participants without diabetes**				
Gender				
	Male (n = 45)	9.13 (7.73)	*t*(97) = -.569	= .570
	Female (n = 54)	8.28 (7.20)		
Total sample (N = 99)		8.67 (7.42)		
Age		41.77 (12.50)	*r* = .065	= .521

Note:

^a)^ Based on nonparametric Kruskal-Wallis H Test because of unequal group sizes

We have also tested the differences and associations between participant’s characteristics and presence of MDD as well as MDD and/or dysthymia (see Table 3). The groups with and without MDD as well as MDD and/or dysthymia were not different with regard to gender, age, education level and diabetes duration.

**Table 3:** Associations between participants’ characteristics and MDD as well as MDD and/or dysthymia categories.

**MDD**					
		with MDD	without MDD	*statistics*	*p value*
		*N*(%)			
Gender					
	Male	25 (7.9%)	135 (42.9%)	χ^2^(1) = 0.760 φ = -0.049	= .383
	Female	30 (9.5%)	125 (39.7%)		
Education level					
	No formal	-	-	χ^2^(2) = 4.588 V = 0.146	= .101
	Some/completed primary school	1 (0.5%)	18 (8.3%)		
	Some/completed secondary school	26 (12%)	114 (52.8%)		
	Higher education (college, post-grad/professional)	5 (2.3%)	52 (24.1%)		
Age		M_rank_	M_rank_		
		107.01	109.58	U = 4018.50 ^a^^)^ Z = -0.252	= .80
Diabetes duration					
		126.84	105.31	U = 2357.00 ^a^^)^ Z = -1.803	= .07
**MDD and/or dysthymia**					
		with MDD and/or dysthymia	without MDD and/or dysthymia	*statistics*	*p value*
		N(%)			
Gender					
	Male	27 (8.6%)	133 (42.2%)	χ^2^(1) = 0.995 φ = -0.056	= .318
	Female	33 (10.5%)	122 (38.7%)		
Education level					
	No formal	-	-	χ^2^(2) = 2.933 V = 0.117	= .231
	Some/completed primary school	1 (0.5%)	18 (8.3%)		
	Some/completed secondary school	26 (12%)	114 (52.8%)		
	Higher education (college, post-grad/professional)	7 (3.2%)	50 (23.1%)		
Age ^a^^)^		M_rank_	M_rank_		
		104.66	110.40	U = 4116.00 ^a^^)^ Z = -0.579	= .562
Diabetes duration					
		125.18	105.38	U = 2527.00^a^^)^ Z = -1.698	= .089

Note:

^a)^ Based on nonparametric Mann–Whitney U test because of unequal group sizes

### 3.2. Reliability and validity of the PHQ-9

We assessed the reliability of the PHQ-9 scale by calculating Cronbach’s alpha reliability coefficients separately for patients with T2DM and the comparison group. Cronbach’s alpha for the Polish version of this tool yielded 0.858 and 0.883, respectively, for these groups. Thus internal consistency of the PHQ-9 is satisfactory, indicating a homogeneous structure of the measure.

In terms of convergent validity, the PHQ-9 scores indicated a strong significant positive correlation with the HDRS (patients with diabetes: *r* = 0.781. *p* < .001; group without diabetes: *r* = 0.846. p < .001; whole group: *r* = 0.882, *p <* .001).

### 3.3. Screening accuracy for major depressive disorder

In the first step we performed logistic regression analysis for patients with T2DM. The analysis indicated that the model containing the PHQ-9 as a predictive factor for current MDD was statistically significant, namely χ^2^(1) = 99.183; *p <* .001. The Hosmer–Lemeshow test indicated goodness of fit of the prediction model (H-L χ^2^(6) = 6.51; *p =* .369). The results indicated that approximately 65% of variability in MDD was explained by the PHQ-9 scores in this group (Nagelkerke’s *R*^*2*^ = 0.65). The Wald test showed that the PHQ-9 score was a significance predictor of the prevalence of MDD, *W*(1) = 41.42; *p <* .001 (OR = 1.588; 95%CI: 1.380–1.829).

**Fig 1:**
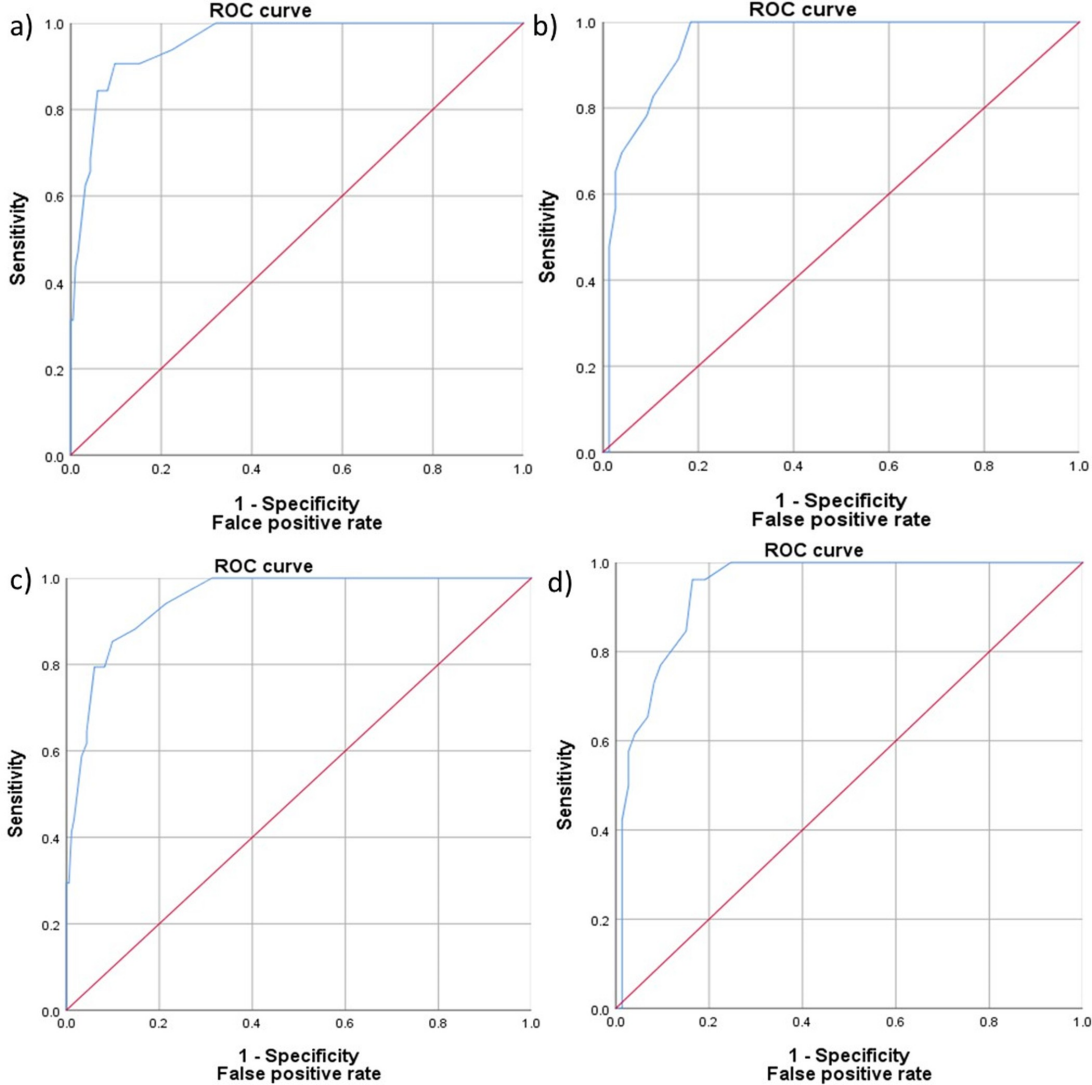
ROC curve of the PHQ-9 for detecting a likely: a) ‘major depressive disorder’ in adults with Type 2 diabetes (N = 216); b) ‘major depressive disorder’ in the non-diabetic group (N = 99); c) ‘MDD and/or dysthymia’ in the diabetic group (N = 216); d) ‘MDD and/or dysthymia’ in the non-diabetic group (N = 99).

The ROC curve was mapped (see Fig 1A). The AUC was 0.956; *p <* .001 (95%CI 0.926–0.985). Youden’s index (0.802) indicated that a cut-off of ≥ 7 yielded the best sensitivity/specificity trade-off: sensitivity 90.62%; specificity 90.22%; PPV 61.7%; and NPV 98.2% (see Table 4). A cut-off score of ≥ 5 was appropriate for a two-stage screening approach for ‘major depressive disorder’ in the diabetic group (sensitivity 93.75; specificity 77.72).

**Table 4:** Accuracy of the PHQ-9 cut-off values for detecting major depression (diagnosed with the MINI) in adults with type 2 diabetes for the Polish sample (N = 216).

PHQ-9 raw score cut-off	Sensitivity	Specificity	Positive Predictive Value	Negative Predictive Value	Positive likelihood ratio	Negative likelihood ratio	Youden's index
≥ 0	1.00	0.00	1.00	0.86	1.00	0.00	0.00
≥ 1	1.00	0.20	0.18	1.00	1.24	0.00	0.20
≥ 2	1.00	0.36	0.21	1.00	1.57	0.00	0.36
≥ 3	1.00	0.55	0.28	1.00	2.24	0.00	0.55
≥ 4	1.00	0.68	0.35	1.00	3.12	0.00	0.68
**≥ 5**^b^^**)**^	**0.94**	**0.78**	**0.42**	**0.99**	**4.21**	**0.08**	**0.71**
≥ 6	0.91	0.85	0.51	0.98	5.96	0.11	0.75
**≥ 7**^a^^**)**^	**0.91**	**0.90**	**0.62**	**0.98**	**9.26**	**0.10**	**0.81**
≥ 8	0.84	0.92	0.64	0.97	10.35	0.17	0.76
≥ 9	0.84	0.94	0.71	0.97	14.11	0.17	0.78
**≥ 10**^c^^**)**^	**0.69**	**0.96**	**0.73**	**0.95**	**15.81**	**0.33**	**0.64**
≥ 11	0.66	0.96	0.72	0.94	15.09	0.36	0.61
≥ 12	0.63	0.97	0.77	0.94	19.17	0.39	0.59
≥ 13	0.47	0.98	0.83	0.91	28.75	0.54	0.45
≥ 14	0.44	0.99	0.88	0.91	40.25	0.57	0.43
≥ 15	0.31	0.99	0.91	0.89	57.50	0.69	0.31
≥ 16	0.31	0.99	0.91	0.89	57.50	0.69	0.31
≥ 17	0.31	1.00	1.00	0.89	- ^(^*^)^	0.69	0.31
≥ 18	0.31	1.00	1.00	0.89	-	0.69	0.31
≥ 19	0.25	1.00	1.00	0.88	-	0.75	0.25
≥ 20	0.16	1.00	1.00	0.87	-	0.84	0.16
≥ 21	0.06	1.00	1.00	0.86	-	0.94	0.06
≥ 22	0.06	1.00	1.00	0.86	-	0.94	0.06
≥ 23	0.06	1.00	1.00	0.86	-	0.94	0.06
≥ 24	0.06	1.00	1.00	0.86	-	0.94	0.06
≥ 25	0.03	1.00	1.00	0.86	-	0.97	0.03
≥ 26	0.03	1.00	1.00	0.86	-	0.97	0.03
= 27	0.03	1.00	1.00	0.86	-	0.97	0.03

Note.

(*) If the sample sizes in the positive (Disease present) and the negative (Disease absent) groups do not reflect the real prevalence of the disease, then the Positive and Negative predicted values, and Accucary, cannot be estimated and you should ignore those values.

a) Optimal cut-off scores according to maximal Youden Index (sensitivity+specificity−1).

b) Recommended cut-off scores for a two-stage screening (maximal sensitivity and ≥75% specificity).

c) Generally recommended cut-off score.

In the next step we conducted logistic regression analysis for the group without diabetes. The PHQ-9 total score was statistically significant as a predictor of current MDD (χ^2^(1) = 58.183; *p <* .001), with the prediction model being a good fit for the data (H-L χ^2^(6) = 1.647; *p =* .949). Approximately 67% of variability in MDD was explained by the PHQ-9 scores (Nagelkerke’s *R*^*2*^ = 0.67). The prevalence of MDD was significantly predicted by the PHQ-9 score, *W*(1) = 22.46; *p <* .001 (OR = 1.438; 95%CI: 1.237–1.671).

The AUC (Fig 1B) was 0.952; *p <* .001 (95%CI 0.913–0.991). Youden’s index (0.82) indicated that a cut-off of ≥ 11 yielded the best diagnostic effectiveness: sensitivity 100% and specificity 81.58% (see Table 5). A cut-off point of ≥ 10 was selected by the two-stage screening approach (sensitivity 100%; specificity 78.95).

**Table 5:** Accuracy of the PHQ-9 cut-off values for detecting major depression (diagnosed with the MINI) in adults without diabetes for the Polish sample (N = 99).

PHQ-0 raw score cut-off	Sensitivity	Specificity	Positive Predictive Value	Negative Predictive Value	Positive likelihood ratio	Negative likelihood ratio	Youden's index
≥ 0	0.00	1.00	0.00	0.77	- ^(^*^)^	1.00	0.00
≥ 1	1.00	0.22	0.28	1.00	1.29	0.00	0.22
≥ 2	1.00	0.26	0.29	1.00	1.36	0.00	0.26
≥ 3	1.00	0.32	0.31	1.00	1.46	0.00	0.32
≥ 4	1.00	0.39	0.33	1.00	1.65	0.00	0.39
≥ 5	1.00	0.46	0.36	1.00	1.85	0.00	0.46
≥ 6	1.00	0.59	0.43	1.00	2.45	0.00	0.59
≥ 7	1.00	0.64	0.46	1.00	2.81	0.00	0.64
≥ 8	1.00	0.67	0.48	1.00	3.04	0.00	0.67
≥ 9	1.00	0.72	0.52	1.00	3.62	0.00	0.72
**≥ 10** ^b^^**),**^ ^c^^**)**^	**1.00**	**0.79**	**0.59**	**1.00**	**4.75**	**0.00**	**0.79**
**≥ 11** ^a^^**)**^	**1.00**	**0.82**	**0.62**	**1.00**	**5.43**	**0.00**	**0.82**
≥ 12	0.91	0.84	0.64	0.97	5.78	0.10	0.76
≥ 13	0.83	0.89	0.70	0.94	7.85	0.19	0.72
≥ 14	0.78	0.91	0.72	0.93	8.50	0.24	0.69
≥ 15	0.74	0.93	0.77	0.92	11.23	0.28	0.67
≥ 16	0.70	0.96	0.84	0.91	17.62	0.32	0.66
≥ 17	0.65	0.97	0.88	0.90	24.78	0.36	0.63
≥ 18	0.61	0.97	0.88	0.89	23.13	0.40	0.58
≥ 19	0.57	0.97	0.87	0.88	21.48	0.45	0.54
≥ 20	0.48	0.99	0.92	0.86	36.35	0.53	0.47
≥ 21	0.43	0.99	0.91	0.85	33.04	0.57	0.42
≥ 22	0.22	0.99	0.83	0.81	16.52	0.79	0.20
≥ 23	0.22	0.99	0.83	0.81	16.52	0.79	0.20
≥ 24	0.17	0.99	0.80	0.80	13.22	0.84	0.16
≥ 25	0.00	0.99	0.00	0.77	0.00	1.01	-0.01
≥ 26	0.00	0.99	0.00	0.77	0.00	1.01	-0.01
= 27	0.00	0.99	0.00	0.77	0.00	1.01	-0.01

Note.

(*) If the sample sizes in the positive (Disease present) and the negative (Disease absent) groups do not reflect the real prevalence of the disease, then the Positive and Negative predicted values, and Accuracy, cannot be estimated and you should ignore those values.

a) Optimal cut-off scores according to maximal Youden Index (sensitivity+specificity−1).

b) Recommended cut-off scores for a two-stage screening (maximal sensitivity and ≥75% specificity).

c) Generally recommended cut-off score.

### 3.4. Screening accuracy for MDD and/or dysthymia

The PHQ-9 total score was a statistically significant predictor of MDD and/or dysthymia, χ^2^(1) = 96.745; *p <* .001. The Hosmer–Lemeshow test indicated goodness of fit of the prediction model, H-L χ^2^(6) = 5.436; *p =* .489. Approximately 62% of variability in ‘MDD and/or dysthymia’ was explained by the PHQ-9 scores (Nagelkerke’s *R*^*2*^ = 0.62). The contribution of the PHQ-9 (predictor) to the prevalence of MDD and/or dysthymia was significant (*W*(1) = 43.137; *p <* .001; OR = 1.552; 95%CI: 1.361–1.769).

The ROC curve is presented in Fig 1C). The AUC was 0.950; *p <* .001 (95%CI 0.920–0.980). Youden’s index (0.75) indicated that a cut-off score of ≥ 7 yielded the best diagnostic effectiveness: sensitivity 85.29% and specificity 90.11% (see Table 6). A cut-off of ≥ 5 points was selected by the two-stage screening approach (sensitivity 94.12%; specificity 78.57%).

**Table 6:** Accuracy of the PHQ-9 cut-off values for detecting any depression disorder (diagnosed with the MINI) in adults with diabetes for the Polish sample (N = 216).

PHQ-9 raw score cut-off	Sensitivity	Specificity	Positive Predictive Value	Negative Predictive Value	Positive likelihood ratio	Negative likelihood ratio	Youden's index
≥ 0	1.00	0.00	0.16	- ^(^*^)^	1.00	- ^(^*^)^	0.00
≥ 1	1.00	0.20	0.19	1.00	1.25	0.00	0.20
≥ 2	1.00	0.37	0.23	1.00	1.58	0.00	0.37
≥ 3	1.00	0.56	0.30	1.00	2.28	0.00	0.56
≥ 4	1.00	0.69	0.37	1.00	3.19	0.00	0.69
**≥ 5** ^b^^**)**^	**0.94**	**0.79**	**0.45**	**0.99**	**4.39**	**0.07**	**0.73**
≥ 6	0.88	0.85	0.53	0.97	5.95	0.14	0.73
**≥ 7** ^a^^**)**^	**0.85**	**0.90**	**0.62**	**0.97**	**8.62**	**0.16**	**0.75**
≥ 8	0.79	0.92	0.64	0.96	9.64	0.22	0.71
≥ 9	0.79	0.94	0.71	0.96	13.14	0.22	0.73
**≥ 10** ^c^^**)**^	**0.65**	**0.96**	**0.73**	**0.94**	**14.72**	**0.37**	**0.60**
≥ 11	0.62	0.96	0.72	0.93	14.05	0.40	0.57
≥ 12	0.59	0.97	0.77	0.93	17.84	0.43	0.56
≥ 13	0.44	0.98	0.83	0.90	26.76	0.57	0.42
≥ 14	0.41	0.99	0.88	0.90	37.47	0.59	0.40
≥ 15	0.29	0.99	0.91	0.88	53.53	0.71	0.29
≥ 16	0.29	0.99	0.91	0.88	53.53	0.71	0.29
≥ 17	0.29	1.00	1.00	0.88	- ^(^*^)^	0.71	0.29
≥ 18	0.29	1.00	1.00	0.88	-	0.71	0.29
≥ 19	0.24	1.00	1.00	0.88	-	0.76	0.24
≥ 20	0.15	1.00	1.00	0.86	-	0.85	0.15
≥ 21	0.06	1.00	1.00	0.85	-	0.94	0.06
≥ 22	0.06	1.00	1.00	0.85	-	0.94	0.06
≥ 23	0.06	1.00	1.00	0.85	-	0.94	0.06
≥ 24	0.06	1.00	1.00	0.85	-	0.94	0.06
≥ 25	0.03	1.00	1.00	0.85	-	0.97	0.03
≥ 26	0.03	1.00	1.00	0.85	-	0.97	0.03
= 27	0.03	1.00	1.00	0.85	-	0.97	0.03

Note.

(*) If the sample sizes in the positive (Disease present) and the negative (Disease absent) groups do not reflect the real prevalence of the disease, then the Positive and Negative predicted values, and Accuracy, cannot be estimated and you should ignore those values.

a) Optimal cut-off scores according to maximal Youden Index (sensitivity+specificity−1).

b) Recommended cut-off scores for a two-stage screening (maximal sensitivity and ≥75% specificity).

c) Generally recommended cut-off score.

Logistic regression analysis indicated that the model including the PHQ-9 total score as a predictor for ‘MDD and/or dysthymia’ was statistically significant (χ2(1) = 57.62; *p <* .001) and the prediction model had a good fit with the data (H-L χ^2^(6) = 4.80; p = .57). Approximately 65% of variability in ‘MDD and/or dysthymia’ was explained by the PHQ-9 scores in the group without diabetes (Nagelkerke’s *R*^*2*^ = 0.646, *W*(1) = 24.15; *p <* .001; OR = 1.401; 95%CI: 1.225–1.603).

The operating characteristics for the diagnosis of ‘MDD and/or dysthymia’ in the group without diabetes are shown in Fig 1D and Table 7. The AUC was 0.942; p < .001 (95%CI 0.898–0.985). For the PHQ-9, an optimal cut-off score of ≥ 11 (sensitivity = 96.0%, specificity = 83.56%) was equal to the cut-off score suggested for Youden’s index (YI = 0.80) and of ≥9 (sensitivity = 100%; specificity = 75.34%) for two-stage screening for ‘MDD and/or dysthymia’.

**Table 7:** Accuracy of the PHQ-9 cut-off values for detecting any depression disorder (diagnosed with the MINI) in adults without diabetes for the Polish sample (N = 99).

PHQ-9 raw score cut-off	Sensitivity	Specificity	Positive Predictive Value	Negative Predictive Value	Positive likelihood ratio	Negative likelihood ratio	Youden's index
≥ 0	1.00	0.00	0.26	- ^(^*^)^	1.00	- ^(^*^)^	0.00
≥ 1	1.00	0.23	0.32	1.00	1.30	0.00	0.23
≥ 2	1.00	0.27	0.33	1.00	1.38	0.00	0.27
≥ 3	1.00	0.33	0.35	1.00	1.49	0.00	0.33
≥ 4	1.00	0.41	0.38	1.00	1.70	0.00	0.41
≥ 5	1.00	0.48	0.41	1.00	1.92	0.00	0.48
≥ 6	1.00	0.62	0.48	1.00	2.61	0.00	0.62
≥ 7	1.00	0.67	0.52	1.00	3.04	0.00	0.67
≥ 8	1.00	0.70	0.54	1.00	3.32	0.00	0.70
**≥ 9** ^b^^**)**^	**1.00**	**0.75**	**0.59**	**1.00**	**4.06**	**0.00**	**0.75**
**≥ 10** ^c^^**)**^	**0.96**	**0.81**	**0.64**	**0.98**	**5.01**	**0.05**	**0.77**
**≥ 11** ^a^^**)**^	**0.96**	**0.84**	**0.68**	**0.98**	**5.85**	**0.05**	**0.80**
≥ 12	0.85	0.85	0.67	0.94	5.62	0.18	0.70
≥ 13	0.77	0.90	0.74	0.92	8.02	0.26	0.67
≥ 14	0.73	0.92	0.76	0.91	8.89	0.29	0.65
≥ 15	0.65	0.93	0.77	0.88	9.55	0.37	0.59
≥ 16	0.62	0.96	0.84	0.88	14.97	0.40	0.57
≥ 17	0.58	0.97	0.88	0.87	21.06	0.43	0.55
≥ 18	0.54	0.97	0.88	0.86	19.65	0.47	0.51
≥ 19	0.50	0.97	0.87	0.85	18.25	0.51	0.47
≥ 20	0.42	0.99	0.92	0.83	30.88	0.58	0.41
≥ 21	0.38	0.99	0.91	0.82	28.08	0.62	0.37
≥ 22	0.19	0.99	0.83	0.77	14.04	0.82	0.18
≥ 23	0.19	0.99	0.83	0.77	14.04	0.82	0.18
≥ 24	0.15	0.99	0.80	0.77	11.23	0.86	0.14
≥ 25	0.00	0.99	0.00	0.73	0.00	1.01	-0.01
≥ 26	0.00	0.99	0.00	0.73	0.00	1.01	-0.01
= 27	0.00	0.99	0.00	0.73	0.00	1.01	-0.01

Note.

(*) If the sample sizes in the positive (Disease present) and the negative (Disease absent) groups do not reflect the real prevalence of the disease, then the Positive and Negative predicted values, and Accuracy, cannot be estimated and you should ignore those values.

a) Optimal cut-off scores according to maximal Youden Index (sensitivity+specificity−1).

b) Recommended cut-off scores for a two-stage screening (maximal sensitivity and ≥75% specificity).

c) Generally recommended cut-off score.

## 4. Discussion

The main aims of this study were to assess both the reliability and validity of the Polish version of the PHQ-9 in patients with and without T2DM and determine the optimal cut-off point for recognizing a disorder of major depression or of MDD and/or dysthymia for people with and without T2DM. To our knowledge, this is the first such study to identify both the psychometric and screening properties of the PHQ-9 simultaneously in these two groups.

The results of our analysis provide empirical evidence for the internal consistency, reliability and convergent validity of the Polish version of the PHQ-9, with a high Cronbach’s alpha and expected strong positive correlation with the HDRS in both groups of people with and without T2DM.

To our knowledge this is the first attempt to indicate criterion validity for the PHQ-9 in order to identify both MDD and MDD and/or dysthymia in people with T2DM. This issue is critical as there is a need for improved recognition and treatment of depressive symptoms in order to prevent severe depression among patients and reduce treatment-related costs [57]. In previous research it was shown that among individuals with dysthymia, the risk of MDD within a year was 5.5 times more likely [58]. The stepped care model for the treatment of depression in diabetic patients assumes that the first step to take for patients with MDD and/or dysthymia identified as being at the highest risk of major depression is to monitor their symptoms and then re-evaluate their mental health [59].

The operating characteristics of the screening instrument for ‘MDD and/or dysthymia’ were very similar to those for ‘MDD’, however, the first ROCs were slightly lower for both the non-diabetic group and for patients with diabetes. One possible reason for this may be that the diagnostic criteria for ‘MDD and/or dysthymia’ are more heterogeneous than those for and they may provide a diagnostic challenge [45].

In our research, the optimal cut-offs for PHQ-9 in people with and without diabetes were investigated based on two methods: 1) a one-step method using Youden’s index [55]; the cut-off points based on this method can be useful in scientific research where the results are aimed to estimate depression prevalence rates and have no impact on clinical decisions; 2) a second method of two-stage screening for depressive disorders to provide guidance for clinical practice. According to this approach, cut-off scores demonstrating maximal sensitivity and specificity of ≥75% are appropriate [45, 56]. The ROC analysis supports the use of the PHQ-9 as a screening tool for verifying likely current MDD and any depression in people with Type 2 diabetes and in a non-diabetic group.

Youden’s index indicated that a cut-off of ≥ 7 yielded the best sensitivity/specificity trade-off and it is useful for screening MDD for research purpose among people with diabetes. On the other hand, the results obtained using the two-stage method show that the cut-off score of ≥ 5 was appropriate to screen for MDD in clinical practice among people with diabetes. Thus, for MDD, both the optimal cut-off score indicated by Youden’s index (≥ 7) and the two-stage (≥ 5) cut-off scores were lower than the generally recommended cut-off score of ≥ 10 (sensitivity = 68.75%, specificity = 95.65%). Similar results were obtained in a study carried out among patients with coronary artery disease where a PHQ-9 cut-off score of ≥10 was 54% sensitive and 90% specific [45]. Among elderly hospitalized patients, a > 6 score as the cut-off point was indicated (sensitivity 70.4% and specificity 78.2%) [46]. In a study of patients with diabetes, Khamseh [60] looked for utilizing the PHQ-9 in screening for depression in Iran. A cut-off score for the PHQ-9 of ≥ 13 provided an optimal balance between sensitivity (73.80%) and specificity (76.20%). High sensitivity is more important for screening purposes than high specificity, and the low sensitivity of a cut-off score of ≥10 makes it inappropriate for this objective.

Youden’s index indicated that a cut-off score of ≥ 11 yielded the best diagnostic effectiveness in scientific research and a ≥ 10 score was selected by the two-stage screening approach as adequate for screening for MDD among people without diabetes in clinical practice. Hence, for this group, the cut-off scores are very similar to the generally recommended cut-off score of ≥10.

Concerning recognizing ‘MDD and/or dysthymia’ in the group with diabetes, the one-step approach indicated that a cut-off score of ≥ 7 yielded the best diagnostic effectiveness for scientific purpose. Our analyses of the PHQ-9 showed that a cut-off score of ≥ 5 was appropriate for clinical practice to screen for ‘MDD and/or dysthymia’ in people with diabetes according to the two-stage method. This is consistent with the recommended threshold demarcating the lower limits of mild depression [28, 29]. For the PHQ-9, an optimal cut-off score of ≥ 11 was equal to the cut-off score in scientific researches suggested by Youden’s index and of ≥ 9 in clinical practice according to the two-stage method for screening ‘MDD and/or dysthymia’ in the non-diabetic group.

Therefore, the cut-off scores for both MDD and ‘MDD and/or dysthymia’ are lower for people with diabetes as compared to the group without diabetes. To our knowledge, this is the first study that applied Youden’s index and a two-stage approach to find the optimal cut-off values among people with diabetes, thus making an important contribution to existing studies in which discrepancies in sensitivity and specificity for the conventional cut-off score of ≥ 10 have been found up to now [45]. Our research compared the PHQ-9 with a structured diagnostic interview, i.e. depression was diagnosed using DSM-IV criteria assessed by a structured interview (MINI), which is considered the gold standard.

This research provides support for the Polish version of the PHQ-9 that may be useful for both clinical practice and empirical research on people with diabetes, as both have satisfactory psychometric properties.

It is important to note that the previous analyses of the Polish version of the questionnaire [43], suggested an optimal cut off point for depression in healthy people at 12. In our study, non-diabetic subjects had an average PHQ-9 score of 8.67, markedly below this threshold. A score relatively high, yet below the threshold for depression, is a result which is consistent with the findings of the most robust assessment of the prevalence of mental disorders conducted in Poland: the EZOP Poland study [61]. This study showed the prevalence of MDD (diagnosed by CIDI) in the general population in Poland was 3.0%, whereas the prevalence of individual depressive symptoms ranged up to 40.2%.

Validation of each language version is necessary for clinical practice to ensure that any employed screening instrument is adapted to the patient’s culture, language, and literacy abilities [62]. Thus, future studies should be aimed at establishing appropriate cut-offs for people with other conditions. Indeed, a recent meta-analysis (including 36 studies) indicated that the optimal cut-off score of the PHQ-9 can vary from one population to another (ranging from 4 to 16) [63]. However, it was emphasized that it was difficult to draw any firm conclusions because the cut-off points were selectively reported [63]. The authors suggested that using a uniform threshold might not be adequate with regard to all settings [63]. Moreover, the authors recommended that reporting the data on all cut-off points in the validation studies should be mandatory and most studies did not meet these criteria. Most previous studies have not indicated the approach that was used to obtain an optimal trade-off between sensitivity and specificity [e.g. [29, 39, 41, 43, 46]]. Our study fulfilled these requirements. Note should be taken that the PHQ-9 cannot be considered a sufficient tool for the diagnosis of depression, as screening instruments cannot replace a full clinical examination.

A limitation to our study with regard to patients with diabetes is that most of the participants were inhabitants of urban rather than rural areas, where accessibility to psychological support might differ. Patients in specialist clinics may differ from those in the wider diabetes population with respect to severity of depressive symptoms. In the second study [43], the limitation was the relatively small size of the group, and the sample was not selected by a random sampling. However, the study included random participants, patients with chronic kidney disease and patients of a daily psychiatric ward. The control group includes patients with other, mostly chronic diseases. Thus, a lack of a group consisting of more healthy participants is the limitation of this study. Despite the relatively small group of people, the results obtained in this study are very similar to those obtained in other validation studies and confirm the high psychometric properties of the PHQ-9, thus providing important clinical implications.
